# Case Report: SARS-CoV-2-associated immune dysfunction manifesting as concurrent fulminant type 1 diabetes mellitus and subacute thyroiditis

**DOI:** 10.3389/fmed.2025.1644656

**Published:** 2025-08-18

**Authors:** Wei Fang, Huanping Wang, Lian Zhong, Jie Xu, Hongxia Zhu

**Affiliations:** Department of Endocrinology, Chengdu Shuangliu Hospital of Traditional Chinese Medicine, Chengdu, China

**Keywords:** SARS-CoV-2 infection, fulminant type 1 diabetes mellitus, subacute thyroiditis, cytokine storm, immune injury

## Abstract

**Objectives:**

The association between SARS-CoV-2 infection and endocrine emergencies (such as fulminant type 1 diabetes mellitus and subacute thyroiditis) has received increasing attention. However, concurrent manifestations of these two conditions within a short period of time after infection are exceedingly rare, and the underlying mechanisms and clinical management strategies remain unclear.

**Case presentation:**

A 45-year-old Chinese man developed sudden polydipsia, polyuria, and cervical pain on day 7, within 2 weeks of SARS-CoV-2 infection. The diagnosis of fulminant type 1 diabetes mellitus complicated by subacute thyroiditis (SAT) was confirmed through laboratory investigations (arterial blood gas analysis, C-peptide release test, and thyroid ultrasound) and imaging. Treatments included fluid resuscitation, continuous intravenous insulin infusion (0.1 U/kg/h), and prednisone (30 mg/day). Acidosis was corrected within 48 h, and SAT symptoms resolved by day 8. At the 6-month follow-up, SAT had completely resolved, but pancreatic β-cell function remained absent, necessitating lifelong insulin therapy.

**Conclusion:**

This case suggests that SARS-CoV-2 may induce dual-gland damage through immune injury mediated by angiotensin-converting enzyme 2 receptor and cytokine storms. Clinicians should be vigilant for acute hyperglycemia and neck pain following SARS-CoV-2 infection. Serial monitoring of blood glucose and thyroid-related parameters is essential as early intervention may improve prognosis.

## Highlights

- This case report describes the first documented occurrence of concurrent fulminant type 1 diabetes (pancreatic involvement) and subacute thyroiditis (thyroid involvement) developing within 14 days of SARS-CoV-2 infection. This clinical presentation highlights COVID-19's emerging potential to induce rapid-onset, multi-glandular damage.
- We propose a unified pathogenesis model: SARS-CoV-2 simultaneously attacks both glands through direct ACE2 receptor-mediated viral injury (due to high ACE2 expression in pancreatic β-cells/thyroid) and indirect cytokine storm.
- Combining intravenous insulin (for diabetic ketoacidosis) with corticosteroids (for thyroiditis) safely resolved both emergencies within 48-72 h. It is suggested that the appropriate use of steroids in the case of intravenous insulin use is safe.

## 1 Introduction

Fulminant type 1 diabetes mellitus (FT1DM), the most critical subtype of diabetes mellitus, is characterized by hyperacute destruction of pancreatic β-cells, fulminant progression to ketoacidosis, and markedly elevated pancreatic enzymes (1, 2). Its pathogenesis is closely associated with genetic susceptibility, autoimmunity, and viral infections (3, 4). Notably, since the emergence of the novel coronavirus (SARS-CoV-2) pandemic, multiple studies have indicated that SARS-CoV-2 may directly damage endocrine glands through the angiotensin-converting enzyme 2 (ACE2) receptor or induce a systemic inflammatory storm, leading to multi-organ immune injury (5, 6). Subacute thyroiditis (SAT), a self-limiting thyroid disorder definitively linked to viral infections, typically presents with thyroid pain, transient thyrotoxicosis, and elevated inflammatory markers (7). Its pathological core involves a virus-triggered immune response that mediates thyroid follicular destruction (8). Although both FT1DM and SAT have been reported as potential complications of SARS-CoV-2 infection (9, 10), their concurrent manifestations within a short post-infection period are exceedingly rare.

Herein, we report a rare case of concurrent fulminant type 1 diabetes mellitus and subacute thyroiditis following SARS-CoV-2 infection, potentially linked to infection-associated immune dysregulation such as a cytokine storm. We aim to delineate the temporal sequence of dual-glandular injury, summarize the distinctive clinical features and management challenges, and underscore the imperative for screening endocrine emergencies after SARS-CoV-2 infection.

## 2 Clinical data

### 2.1 Case descriptions

**Figure 1:**
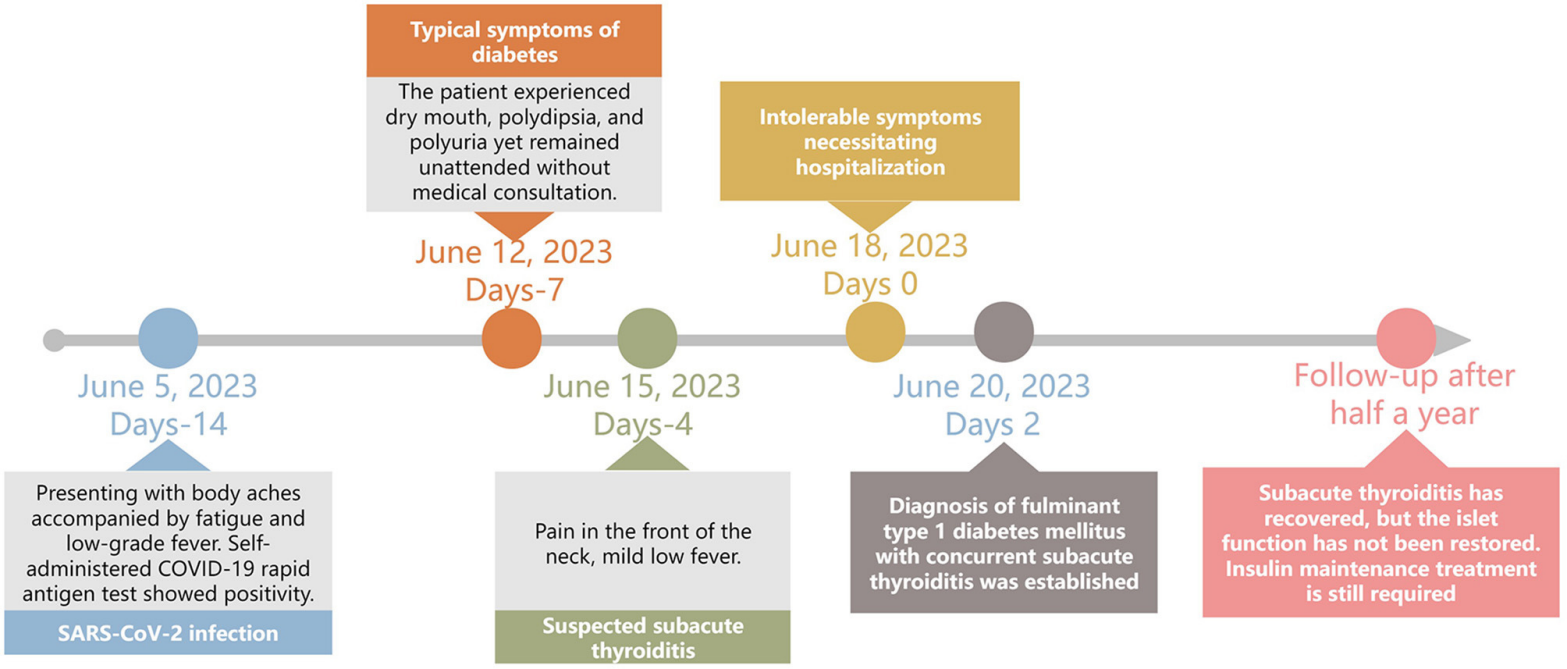
Timeline of the patient's clinical course.

A 45-year-old Chinese man presented with a 2-week history of symptoms. Two weeks before admission (June 5, 2023), he experienced transient myalgia accompanied by fatigue and low-grade fever. The self-administered COVID-19 nucleic acid test results were positive. His symptoms resolved spontaneously within 3 days without any medication. Seven days later, he developed polydipsia, polyuria, and xerostomia, for which he did not seek medical attention. Ten days after the initial symptoms (and 3 days after the onset of polyuria/polydipsia), he reported cervical pain associated with low-grade fever, but yet again did not seek medical care. He was hospitalized with significant nausea, profound fatigue, and intolerable neck pain. His medical history was unremarkable and he specifically denied having diabetes mellitus. There had no history of surgery, trauma, or substance abuse. He reported a moderate history of smoking and alcohol consumption. His father had a history of type 2 diabetes mellitus; however, no significant family history was reported. The patient had not received any COVID-19 vaccination. Figure 1 illustrates the overall clinical course of the patient.

**Figure 2:**
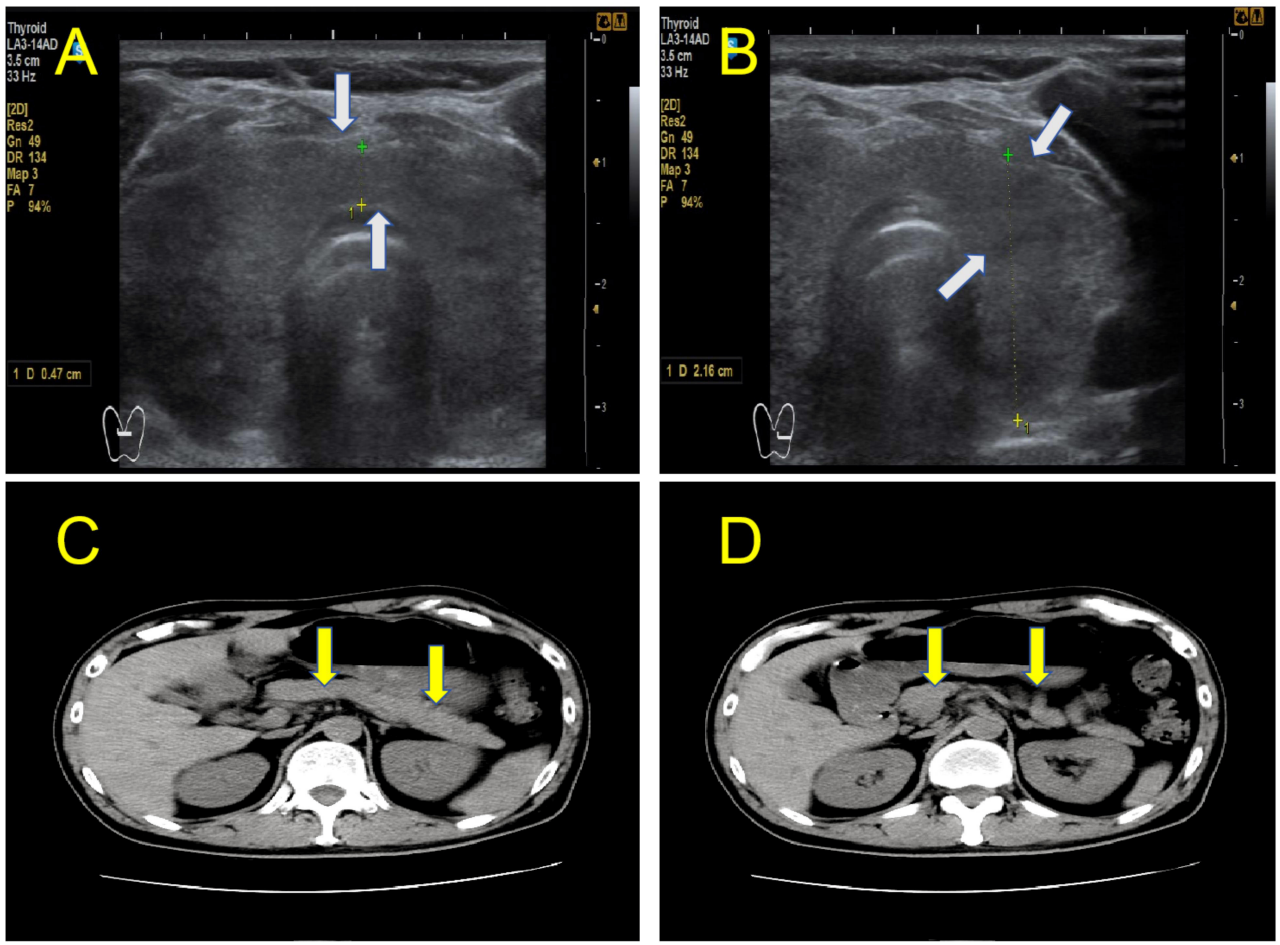
**(A, B)** Ultrasound showed local thyroid enlargement [gray arrow]. **(C, D)** The abdominal CT scan showed no morphological changes of the pancreas [yellow arrow].

On admission, the patient presented with mild dehydration, listlessness, and extreme fatigue but was conscious. Vital signs monitoring showed a body temperature of 37.6°C, heart rate of 108 beats/min, respiratory rate of 21 breaths/min, blood pressure 125/76 mmHg, body mass index (BMI) of 21.2 kg/m^2^, and weight of 65 kg. Physical examination revealed a Grade I enlarged, tender thyroid gland. An abdominal examination revealed mild epigastric tenderness without rebound tenderness or guarding. Laboratory tests including arterial blood gas analysis showed a pH of 6.94, partial pressure of carbon dioxide 28.72 mm Hg, actual bicarbonate 6.0 mmol/L, base excess −25.4 mmol/L, and lactate 5.19 mmol/L; venous serum glucose 22.91 mmol/L, potassium 5.99 mmol/L, creatinine 123 μmol/L, urea 12.3 mmol/L, triglycerides 9.65 mmol/L, cholesterol 8.46 mmol/L; glycated hemoglobin (HbA1c) 6.2%, normal serum amylase, plasma osmolarity 318.19 mOsm/(kg·H_2_O), white blood cells 19.60 × 10^9^/L, erythrocyte sedimentation rate (ESR) 76.2 mm/h, high-sensitivity C-reactive protein 51.8 mg/L, normal thyroid function, and negative thyroid autoantibodies (TPO-Ab, TG-Ab). The tests for other potential viral nucleic acids and antibodies yielded negative results. Thyroid color Doppler ultrasound indicated local thyroid enlargement, suggesting subacute thyroiditis (Figures 2A, B); whereas abdominal computed tomography (CT) showed no pancreatic changes (Figures 2C, D). After the patient's condition stabilized, we conducted an oral glucose tolerance test (OGTT), insulin and C-peptide release tests, and pancreatic autoantibody tests (Supplementary Table 1). The results indicated a complete loss of pancreatic function and absence of pancreatic autoantibodies. The patient's detailed laboratory test values are shown in Supplementary Table 2. Written informed consent was obtained from participant, and the study protocol received approval from the Ethics Committee of Chengdu Shuangliu District Hospital of Traditional Chinese Medicine.

### 2.2 Diagnosis and treatment

The patient's condition was critical. The preliminary diagnoses were diabetic ketoacidosis (DKA) and FT1DM complicated by SAT, and accompanied by significant metabolic derangements (metabolic acidosis, renal dysfunction, and hyperkalemia). Acute pancreatitis-induced hyperglycemia and metabolic disorders were excluded. Acute pancreatitis usually presents with transient hyperglycemia and rarely requires long-term insulin treatment (11). Moreover, pancreatic CT imaging and pancreatic enzyme levels were normal. The patient did not meet the diagnostic criteria for diabetic hyperosmolar state (HHS) (12), which are middle-aged and older patients with a blood glucose ≥ 33.3 mmol/L, effective plasma osmolarity ≥ 320.0 mOsm/(kg·H_2_O), serum bicarbonate ≥ 15 mmol/L or arterial blood gas pH ≥ 7.3, and strong positive urine ketones but negative or weak positive blood ketones. Although we need to be aware of the concurrent occurrence of HHS and DKA, HHS always presents with obvious hyperosmolarity. Stress hyperglycemia is temporary and does not match the patient's persistent hyperglycemia, requiring long-term insulin treatment. Finally, lactic acidosis is common in patients taking phenformin, drinking alcohol, or with a history of underlying diseases such as renal dysfunction, shock, and heart failure, and blood lactate levels >7 mmol/L (13). However, the patient's blood lactate level was only slightly increased and symptoms were rapidly relieved after fluid resuscitation; therefore, this diagnosis was not considered. Regarding subacute thyroiditis, thyroid tumors should be considered. Local pain may occur when tumors bleed, necrotize, or compress nerves, but the patient's B-ultrasound examination did not support this (14). Acute suppurative thyroiditis is a non-specific infection of the thyroid gland, often occurring in the left lobe, and is a local manifestation of systemic sepsis accompanied by systemic sepsis symptoms (15). However, the present patient did not meet these criteria. Hashimoto's thyroiditis can cause thyroid pain and tenderness in some patients, and the ESR may be slightly elevated during the active stage. However, serum TgAb and TPOAb titers increase (16), which does not match the patient's condition. Therefore, the patient was diagnosed FT1DM combined with SAT.

The patient was immediately treated with fluid resuscitation and intravenous insulin infusion. Within the first 24 h, ~6,000 mL of intravenous fluids were administered, and insulin was infused at approximately 0.1 U/kg/h to suppress ketogenesis. When the blood glucose fell below 13.9 mmol/L, glucose-containing fluids were added at the appropriate ratios. The patient had hyperkalemia and abnormal renal function, which were attributed to pre-renal injury secondary to DKA. After the urine output exceeded 40 mL/h and serum potassium normalized, potassium replacement was promptly administered. Concurrently, prednisone tablets 30 mg orally once daily were started, with a weekly reduction of 5 mg. After 48 h, the patient reported significant symptomatic improvement, with resolution of fever and alleviation of neck pain. Arterial blood gas analysis confirmed correction of the acidosis. The hyperkalemia, hyperlipidemia, and renal dysfunction improved. Treatment was transitioned to subcutaneous insulin therapy for intensive glycemic control with a total daily insulin dose of approximately 50 U. Prednisone therapy was continued for anti-inflammatory purposes and fenofibrate was added for lipid control. After 8 days, all symptoms resolved. Electrolyte levels, renal function, lipid profile, and blood gas analysis results normalized, and the patient was discharged. The discharge insulin regimen was insulin aspart 10 U before breakfast, 9 U before lunch, and 9 U before dinner, with insulin glargine 14 U at bedtime. The prednisone dosage was continued with a tapering schedule. At the 6-month follow-up, the patient's subacute thyroiditis had completely resolved, and prednisone was discontinued. However, pancreaticβ-cell function remained absent, necessitating continued insulin replacement therapy.

## 3 Discussion

The patient had no history of diabetes mellitus and had never received a COVID-19 vaccine. The patient abruptly developed DKA during the convalescent phase of COVID-19. Upon admission, the HbA1c level was 6.2%. This presentation meets all three core diagnostic criteria for FT1DM: rapid onset (within ~1 week) of diabetic ketosis or ketoacidosis after the appearance of hyperglycemic symptoms; initial blood glucose ≥ 16.0 mmol/L and HbA1c < 8.5% at diagnosis; and fasting serum C-peptide < 0.10 nmol/L (0.3 ng/mL), and post-glucagon stimulation or postprandial serum C-peptide peak < 0.17 nmol/L (0.5 ng/mL) (17). Concurrently, the patient presented with neck pain, thyroid tenderness, and a significantly elevated ESR. Thyroid ultrasonography revealed thyroid enlargement with decreased vascular flow, definitively supporting the diagnosis of subacute thyroiditis (18). The close temporal association between the two conditions (emerging sequentially within 14 days post-SARS-CoV-2 infection) strongly suggests that SARS-CoV-2 may act as a common triggering factor. It has been proposed that the virus induces synchronous damage to both the pancreas and the thyroid through multifaceted mechanisms.

The precise mechanism by which SARS-CoV-2 induces dual-gland damage remains unclear and likely involves multiple factors. However, direct viral invasion by SARS-CoV-2 and the infection-triggered cytokine storm are likely significant components of the pathogenesis. SARS-CoV-2 invades host cells by binding to ACE2 receptor. Both human pancreatic β-cells and thyroid follicular cells highly express ACE2, providing an anatomical basis for direct viral attack (19, 20). Within pancreatic tissue, SARS-CoV-2 infection can disrupt β-cell membrane structure and induce endoplasmic reticulum stress, leading to the cessation of insulin synthesis. Moreover, SARS-CoV-2 infection can directly induce β-cell lysis and death (21, 22). Concurrently, viral invasion of the thyroid follicles triggers abnormal thyroglobulin release, manifesting as transiently elevated FT4 levels during the acute phase of SAT (23). Autopsy studies have detected viral envelope proteins within pancreatic acinar cells in COVID-19 fatalities, further supporting the direct cytolytic theory (24). SARS-CoV-2 can induce the massive release of pro-inflammatory cytokines such as IL-6 and TNF-α, forming a cytokine “storm” (25, 26). On one hand, these inflammatory cytokines accelerate pancreatic β-cell apoptosis by activating the NF-κB pathway (27)and promote the substantial release of chemokines (e.g., CXCL10), which attract macrophage infiltration into thyroid tissue (28). In contrast, under inflammatory stimulation, thyroid follicles release stored thyroid hormones, leading to transient destructive thyrotoxicosis (29). These inflammatory cascades reveal the temporal pattern of dual-gland damage: during the acute viral infection phase (week 1), the high inflammatory burden combined with ACE2-mediated direct viral attack on β-cells triggers the fulminant course of FT1DM. Upon entering the post-infection immunomodulatory phase (week 2), persistent viral fragments continuously activate immune responses within the thyroid follicles, resulting in the characteristic symptoms of SAT.

Clinically, this dual pathology presents unique management challenges. The primary challenge lies in diagnostic pitfalls; neck pain associated with subacute thyroiditis can easily be misattributed to COVID-19-related lymphadenitis or pharyngeal complications. Nausea, abdominal pain, and other manifestations accompanying FT1DM often overlap with the gastrointestinal symptoms of COVID-19, potentially leading to delayed interventions. At the treatment level, significant contradictions exist; during the thyrotoxic phase of SAT, the use of beta-blockers should be avoided (as they may mask hypoglycemia warning symptoms). However, intensive insulin therapy for FT1DM necessitates strict prevention of blood glucose fluctuations. Although glucocorticoids can effectively alleviate local inflammation in SAT, they can also exacerbate insulin resistance (30). This forces clinicians to achieve a nuanced balance between glycemic control and pain management as SAT typically follows a self-limiting course, whereas FT1DM requires lifelong insulin replacement therapy. Notably, patients developing FT1DM post-COVID-19 exhibit more profound islet function failure (5). Furthermore, these patients may have an elevated risk of cardiovascular events (31).

We searched PubMed, Web of Science, and MEDLINE databases for reported cases of SARS-CoV-2 infection-associated FT1DM and/or SAT. Only three individual case reports of FT1DM following SARS-CoV-2 infection have been documented (32–34), the details of which are shown in Table 1, whereas over hundred case series reports of SAT exist (35, 36). However, the concurrent presentation of these two conditions has not been previously reported, making this the first documented case of SARS-CoV-2-associated FT1DM co-occurring with SAT.

**Table 1:** Comparison of characteristics of FT1DM after SARS-CoV-2 infection.

	**Our case**	**Case 1 Pan et al. (32)**	**Case 2 Zhou et al. (33)**	**Case 3 Wang et al. (34)**
Age of onset (years)	45	46	34	42
Sex	Male	Woman	Woman	Male
Vaccination history	None	Yes	Yes	Yes
Pregnancy	-	None	Yes	-
Gradient of infection	Mild	Mild	Mild	Mild
The time from viral infection to the occurrence of FT1DM	2 weeks	12 Days	5 weeks	5 weeks
Symptoms	Thirst, excessive drinking, severe fatigue	Thirst, excessive drinking, upper abdominal pain	Excessive drinking, nausea and vomiting	Thirst, polyuria and weight loss
BMI, kg/m^2^	21.2	22.3	21	26.6
Plasma glucose levels, mg/dL	22.91 mmol/L	34.37 mmol/L	29.0 mmol/L	21.4 mmol/L
Serum pancreatic enzyme	Normal	Normal	Mildly elevated	Normal
CT/MRI findings	Normal (CT)	Normal (MRI)	Normal (CT)	N
Therapy	Insulin and fluid replacement	Insulin and fluid replacement	Insulin, fluid resuscitation, low-dose sodium bicarbonate	Insulin and fluid replacement
Whether it is antiviral treatment	None	None	None	None
HbA1c (%) at onset of FT1DM	6.2%	6.7%	5.9%	7.3%
C-peptide (ng/ml)	< 0.003	0.19	0.02	0.14
Islet autoantibodies	Negative	Negative	Negative	Negative
Outcome	Improved	Improved	Improved	Improved

N, not provide; FT1DM, Fulminant type 1 diabetes; BMI, body mass index; CT, computerized tomography; MRI, magnetic resonance imaging.

Among the reported cases of SARS-CoV-2-associated FT1DM, including the present case, the age of onset ranges from 34 to 46 years. Two patients were women and two were men. All four developed DKA within 7 days of symptom onset. Their body mass indices were within normal limits and islet-associated autoantibodies were negative, consistent with the clinical features of FT1DM (37, 38). Notably, pancreatic imaging changes and significant alterations in pancreatic enzymes were absent in all the cases. This contrasts with previous reports on FT1DM (39), suggesting that SARS-CoV-2-associated FT1DM primarily affects the pancreatic endocrine system. Conversely, SARS-CoV-2-associated SAT cases predominantly involved women. Clinical presentations could be atypical, and thyroid ultrasonography proved to be crucial for a correct diagnosis. Approximately 50% of ultrasound examinations indicated thyroid enlargement (40), a finding consistent with the ultrasonographic results in the present case.

However, this case report has inherent limitations. We could not establish a direct causal relationship between SARS-CoV-2 infection and the development of FT1DM or SAT. Future studies are required to establish multicenter cohorts to elucidate the epidemiological characteristics of such complications and to further investigate the underlying molecular mechanisms and genetic backgrounds.

## 4 Conclusion

This study reports a rare case of concurrent FT1DM and SAT, highlighting a pathological pattern where viral infection potentially acts as a common trigger and cytokine storms lead to dual-gland damage. The diagnosis and treatment of SARS-CoV-2-associated FT1DM combined with SAT present significant challenges and contradictions. DKA resulting from FT1DM is life threatening. Timely diagnosis and intervention are crucial to improve patient prognosis. Clinicians should be vigilant of acute hyperglycemic symptoms or neck pain that occur after SARS-CoV-2 infection. We recommend routine screening for blood glucose levels, pancreatic enzymes, thyroid function, and inflammatory markers in such patients.

## Data Availability

The original contributions presented in the study are included in the article/Supplementary material, further inquiries can be directed to the corresponding authors.
