# Supplementary material for: Case Report: SARS-CoV-2-associated immune dysfunction manifesting as concurrent fulminant type 1 diabetes mellitus and subacute thyroiditis
Source: Front Med (Lausanne). 2025 Aug 18;12:1644656. doi: 10.3389/fmed.2025.1644656 (PMC12401013; doi:10.3389/fmed.2025.1644656)
Supplement: Supplementary file 1 [file Data_Sheet_1.pdf]

Supplementary Material

Case report: SARS-CoV-2-associated immune dysfunction manifesting as concurrent fulminant type 1 diabetes mellitus and subacute thyroiditis

Wei Fang[1†], Huanping Wang[1†*],Lian Zhong[1], Jie Xu[1], Hongxia Zhu[1*]

[1]Department of endocrinology, Chengdu Shuangliu Hospital of Traditional Chinese Medicine, Chengdu, China.

*Correspondence:Hongxia Zhu, 2780019047@qq.com. Huanping Wang,fangw139@163.com.

[†]These authors contributed equally to this work and share first authorship

Keywords: SARS-CoV-2 infection, fulminant type 1 diabetes mellitus, subacute thyroiditis, cytokine storms, immune injury

Supplementary table.1 Results of pancreas islet function test

Index	OGTT+Insulin release tests+C peptide release tests			
	0 h	1 h	2 h	3h
Blood glucose(mmol/L)	9. 52	21.58	27.30	22.14
Insulin(pmol/L)	16. 80	13.91	13.75	13.33
C peptide (nmol/L)	＜0.01	＜0.01	＜0.01	＜0.01

Abbreviations:OGTT, oral glucose tolerance test.

Supplementary table.2 Laboratory findings

Assessment	Results	Reference
Hematology		
White blood cell count (*10^9/L)	19.6	4.0 – 10.0
Neutrophils (%)	89.54	50.0 – 70.0
Hemoglobin (g/L)	121.0	130.0 – 175.0
Platelet (*10^9/L)	260.0	100.0 – 300.0
High-sensitivity C-reactive protein(mg/L)	51.8	0.0 – 10.0
Biochemistry		
Blood glucose (mmol/L)	22.91	4.11 – 5.89
Hemoglobin A1c (%)	6.20	4.10 – 6.10
Urea (mmol/L)	12.3	2.14 – 7.14
Creatinine (umol/L)	123.0	59 – 104
Na (mmol/L)	135.5	136 – 145
K (mmol/L)	5.99	3.5 – 5.2
Cl (mmol/L)	94.9	96 – 108
Ca (mmol/L)	2.36	2.15 – 2.50
Albumin (g/L)	53.8	35 – 52
AST (U/L)	30.4	0.0 – 40.0
ALT (U/L)	31.3	0.0 – 42.0
Total bilirubin (umol/L)	4.5	2.5 – 21.0
ALP (U/L)	110.0	40.0 – 130.0
TC (mmol/L)	8.46	1.10 – 5.20
TG (mmol/L)	9.65	0.0 – 2.26
Troponin T (pg/mL)	5.06	0.0 – 14.0
Amylases (U/L)	35.0	28.0 – 100.0
Thyroid		
TSH (mIU/L)	3.75	0.4 – 4
FT4 (pmol/L)	13.0	10.3 – 25.74
FT3 (pmol/L)	2.54	3.1-6.8
TG-Ab (U/mL)	0.72	0.0 – 4.11
TPO-Ab (U/mL)	11.2	5.0 – 34.0
Blood gas analysis		
pH	6.94	7.33 – 7.42
PcO_2 (mmHg)	28.72	35.00 – 45.00
HCO3 (mmol/L)	6.0	21.40 – 27.30
Base excess (mmol/L)	-25.4	-2.0 – 3.0
Lactate (mmol/L)	5.19	0.7 – 2.1
Urine		
Urine Microalbumin (mg/L)	-	0.0 – 25.0
Protein	-	-
Glucose	+++	-
Urinary ketone	++++	-
islet autoantibodies		
Glutamic acid decarboxylase antibody（Anti-GAD）	Negative	-
Tyrosine phosphatase antibody（Anti-IA2）	Negative	-
Antibody to zinc transporter 8（Anti-ZnT8）	Negative	-
Anti-islet cell antibody（Anti-ICA）	Negative	-

Insulin antibodies（Anti-IA）	Negative	-
Infection		
Epstein-Barr virus IgM antibody	Negative	-
Coxsackievirus IgM antibody	Negative	-
human immunodeficiency virus antibody(HIV)	Negative	-
Adenovirus	Negative	-
Inflfluenza A virus PCR	Negative	-
Inflfluenza B virus PCR	Negative	-
Respiratory syncytial virus PCR	Negative	-
Parainfluenza virus type I PCR	Negative	-
Parainfluenza virus type III PCR	Negative	-
SARS-CoV-2 PCR	Positive	-

Abbreviations: AST, aspartate aminotransferase; ALT, alanine aminotransferase; ALP, alkaline phosphatase; TC: total cholesterol; TG, triglyceride; TSH, thyroid stimulating hormone; FT4: free-thyroxine; FT3:free triiodothyronine; TG-Ab:thyroglobulin antibodies; TPO-Ab: thyroid peroxidase antibody; pH:Pondus Hydrogenii; PcO2:Partial Pressure of Carbon Dioxide.
